# Media Information Compared to Scientific Studies Regarding Piranha Attacks in Brazil

**DOI:** 10.1590/0037-8682-0213-2025

**Published:** 2025-10-17

**Authors:** Patrícia Tatiane Gomes, Sheila Canevese Rahal, Edson Luiz Fávero, Adriana Lúcia Mendes, Itamar Alves Martins, Vidal Haddad

**Affiliations:** 1 Universidade Estadual Paulista “Júlio de Mesquita Filho”, Faculdade de Medicina Veterinária e Zootecnia, Pós-graduação em Animais Selvagens, Botucatu, SP, Brasil.; 2 Universidade Estadual Paulista “Júlio de Mesquita Filho”, Faculdade de Medicina, Departamento de Clínica Médica, Botucatu, SP, Brasil.; 3 Universidade de Taubaté, Departamento de Biologia, Laboratório de Zoologia, Taubaté, SP, Brasil.; 4 Universidade Estadual Paulista “Júlio de Mesquita Filho”, Faculdade de Medicina, Departamento de Infectologia, Dermatologia, Diagnóstico por Imagem e Radioterapia, Botucatu, SP, Brasil.

**Keywords:** Bites and stings, Traumatogenic fish, Freshwater fish, South American fish

## Abstract

**Background:**

Piranhas are carnivorous fish that inhabit rivers in Central and South America, and they are popularly recognized as relentless hunters of continental waters. Their reputation as killers is fueled by folklore and cinematographic works, which contribute to the creation of myths, generation of fear among people, and the vilification of fish.

**Methods:**

We analyzed several media reports on piranha bites that occurred in Brazil, seeking to demystify these attacks as they are described in the lay press, using the injuries and circumstances observed as a basis. In addition, we highlighted human actions that directly affect piranha behavior**.**

**Results:**

Of the 711 cases reported in humans in the last 10 years, 82.27% were classified as mild, with single “punch-out”-shaped injuries associated with the behavior of males to protect nests and larvae in dams and lakes. Seasonal analysis revealed that 29.62% of attacks occurred during the breeding season and 25.92% were associated with improper disposal of food in rivers.

**Conclusions:**

Media coverage of piranha attacks tends to negatively reinforce popular perceptions of the behavior of these species toward humans, diverting attention from human responsibility for the environmental impacts that directly influence the occurrence of these injuries.

## INTRODUCTION

Piranhas are Neotropical carnivorous fish found in Central and South America. They are found in rivers and lakes in several river basins, being abundant in the Amazon and Orinoco regions and in the Prata and São Francisco River basins^1^ ^-^ ^3^ .

**FIGURE 1:**
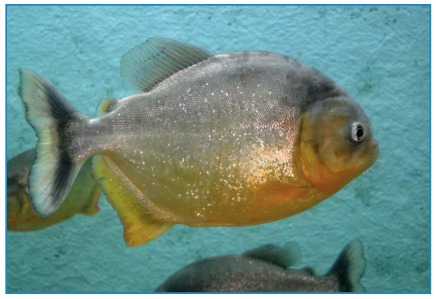
Pirambeba (*Serrasalmus maculatus*): common in dams and rivers of the Southeast region of Brazil.

**FIGURE 2:**
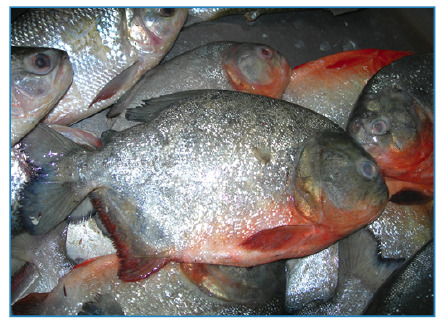
Piranha-caju (*Pygocentrus nattereri*): species frequent in the Midwest and Northern regions.

*Serrasalmus maculatus* (Figure 1), also known as pirambeba, is the most abundant in dammed stretches and streams, and is the main cause of bites in the southeast region of Brazil. *Pygocentrus nattereri* (Figure 2), popularly known as piranhas-caju or cashew-piranhas, is typical of lentic regions and causes injuries in the Central Amazon and Pantanal of the Midwest region^1^ ^-^ ^3^. They are reddish in color, with a grayish head and back^4^.

The reproductive season of *Serrasalmus maculatus* is longer in the upper Paraná River, extending from September (dry season) to January (high season), where environmental conditions favor the emergence of aquatic plants that guarantee spawning sites and serve as shelter for larvae and juveniles^1^ ^,^ ^2^.

During spawning, the couples position themselves side by side, the females then lay eggs on the roots of the aquatic plants so that the males can fertilize them and later care for the offspring^1^ ^,^ ^2^. Some species reach a total length of approximately 50 cm. Piranhas are active mainly during the day and extend their foraging time until the beginning of the night, as they visually orient themselves toward their food^1^ ^-^ ^3^.

The bad reputation of piranhas originated in 1913-1914, with the publication of the book “Through the Brazilian Wilderness” by Theodore Roosevelt, a former American president who embarked on a scientific expedition to explore rivers in the Amazon rainforest^4^. One of Roosevelt’s most famous observations about piranhas was their ability to devour dead or injured animals in a matter of minutes, describing scenes in which piranhas clean the bones of animals in an impressive and frightening manner^4^. The description confirmed the reputation of piranhas as fearsome fish and fueled many myths surrounding these animals, from movies to popular expressions such as the “boi-de-piranha or piranha’s cow”^4^.

Despite this reputation, the species does not hunt in groups, and “feeding frenzy” attacks on large, live prey are rarely observed. The popular reports of piranha attacks on humans is probably due to their scavenging habits, as these fish usually consume dead or injured animals. Reported piranha attacks in the media negatively reinforce the popular idealization of the behavior of these species in relation to humans^5^ ^,^ ^6^.

This study aimed to analyze the information broadcast by the media about piranha attacks that occurred in Brazil, highlight the human actions that influence the defense behavior of this species, and emphasize the importance of carnivorous fish species in natural population control to maintain the balance of aquatic ecosystems. The data presented in this study did not include reports by health systems but focused only on the outbreaks reported in the country by media sources. 

## METHODS

Only states that had injury rates of more than 20 individuals reported in the media between 2012 and 2022 were analyzed. To quantify accidents, keywords were used at research sites and separated by state. The main news websites consulted were Globo Notícias, Conexão Tocantins, I7 Notícias, Portal da Cidade Santa Helena, Assis Notícias, Agência Brasil Comunicações, Folha de Boa Vista, and Redação O Sul. The information was compared with all reports to obtain more accurate data, and organized in an Excel file according to the state, municipality, place of occurrence, number of people injured, characteristics and region of injuries, time of occurrence, time of outbreak, year, and the hypotheses given by “experts.” The Google Earth Pro application was used to evaluate the municipal characteristics as well as capture satellite images of the locations of occurrences and identify the areas in which they occurred and the possible anthropogenic causes. According to the local Human Ethics Committee, images and patients without a clinical history and without facial details do not require authorization from the committee for publication. 

## RESULTS

The states of São Paulo, Alagoas, Amazonas, Bahia, Ceará, Goiás, Minas Gerais, Mato Grosso, Pará, Paraná, Roraima, Rio Grande do Sul, and Tocantins were notified by the media through basic health units, fire departments, and the municipal civil defense (Table 1).

**TABLE 1:** Quantification and characteristics of injuries found in the Brazilian territory.

States	Reported Injuries	Features	Causes
Tocantins	309	Single bites	Dammed water, discarded food
Roraima	140	Single bites	Dammed water, discarded food
São Paulo	78	Single bites	Dammed water, discarded food
Alagoas	68	Single bites	Breeding season
Paraná	40	Single bites	Dammed water, discarded food.

**FIGURE 3:**
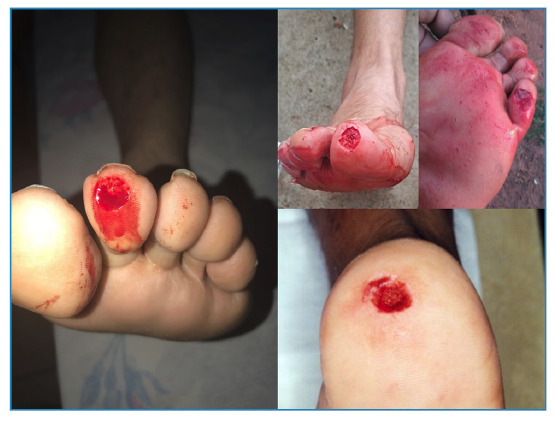
Punch-out shaped lacerations, characterized by a single bite on the leg.

Owing to the teeth of piranhas, the injuries had evident “punch-out” shapes (Figure 3), and the severity varied according to the force of the bite and the body part affected. Almost all accidents reported in the media referred to single bites on the hands, legs, and feet of individuals, ranging from mild to moderate injuries with loss of skin fragments and muscle tissue and rarely loss of bone tissue. From the sources analyzed according to the degree of severity, of the 711 human cases reported in the last 10 years throughout Brazil, 82.27% were considered mild with single bites on the hands and feet with only skin loss, 0.70% were considered serious because of muscle and occasional bone loss, and 0.14% (one case) was considered isolated with the victim found dead with injuries compatible with piranha bites. Approximately 16.87% of these patients did not have the injuries described in the reports. According to local information, the factors that cause conflicts contribute to these attacks. include the breeding season (29.62%), improper disposal of food (25.92%), damming by hydroelectric power plants (UHE) (14.81%), dry season (11.11%), spawning grounds (7.40%), an imbalance in natural predator species (3.70%), damaged protective screens (3.70%), the presence of water hyacinths on riverbanks (3.70%), and young fish learning to hunt (3.70%). Notably, this information was obtained from reports published after the accidents. Although some of the information was presented by individuals identified as experts, no evidence proves that these people were, in fact, experts in the aforementioned area. The seasonality of piranha bites was not fully explained in these reports; many of the reported cases did not specify the day, month, and time of occurrence. However, 33 reports clarified the period of the accidents, while in 7 reports, 332 cases were not clarified.

Despite folklore portraying surreal attacks by schools of piranhas on large animals and humans, no scientific evidence proves these events^7^ ^-^ ^9^. The piranha species *Pygocentrus nattereri* and *Serrasalmus maculatus*, which are mostly associated with the accidents, have opportunistic and scavenging feeding behaviors and can feed on birds, mammals, and fish that are already in the process of decomposition. However, their main diet comprises live fish and whole or fragmented parts of scales, fins, or muscles.

The characteristics of piranha accidents in the Brazilian territory as described in the media include isolated circular shaped bites that involve the skin and occasionally muscles, which can cause extensive bleeding due to damaged blood vessels^7^ ^-^ ^9^. Most accidents occurred during the reproductive season, which is a warning behavior characteristic of male piranhas to protect their nest and offspring (Table 1).

Piranhas, like all predator species, are extremely important for maintaining the balance and health of ecosystems, as they control populations and feed on sick or dead fish that are decomposing.

## DISCUSSION

Of the 711 reported cases, 3 were considered serious owing to partial amputation of the phalanges, which occurred mainly in children whose phalanges are small. These cases occurred in the states of Goiás (2) and São Paulo in Brazil. In addition, 585 cases were classified as minor with only single bites. Of these, 318 occurred during the spawning season, reinforcing the hypothesis that the accidents were caused by males protecting their offspring. These data estimate that the probability of an accident being considered serious is 0.68%, that is, one case in every 147 cases. All the reports analyzed used the term “attacks” to inform the public about accidents involving piranhas. The profile of accidents caused by piranhas in Brazil is completely different from that reported by the media.

## Data Availability

Research data is available upon request.
